# The crucial role of locus coeruleus noradrenergic neurons in the interaction between acute sleep disturbance and headache

**DOI:** 10.1186/s10194-024-01714-5

**Published:** 2024-03-05

**Authors:** Bozhi Li, Ya Cao, Huijuan Yuan, Zhe Yu, Shuai Miao, Chunxiao Yang, Zihua Gong, Wei Xie, Chenhao Li, Wenhao Bai, Wenjing Tang, Dengfa Zhao, Shengyuan Yu

**Affiliations:** 1 Department of Neurology, the First Medical Center, Chinese PLA General Hospital, Fuxing Road 28, Haidian District, Beijing, 100853 People’s Republic of China; 2 Neurology Institute of Chinese PLA General Hospital, the First Medical Center, Chinese PLA General Hospital, Fuxing Road 28, Haidian District, Beijing, 100853 People’s Republic of China; 3 Medical School of Chinese PLA, Beijing, 100853 People’s Republic of China; 4 School of Medicine, Nankai University, Tianjin, China

## Abstract

**Background:**

Both epidemiological and clinical studies have indicated that headache and sleep disturbances share a complex relationship. Although headache and sleep share common neurophysiological and anatomical foundations, the mechanism underlying their interaction remains poorly understood. The structures of the diencephalon and brainstem, particularly the locus coeruleus (LC), are the primary sites where the sleep and headache pathways intersect. To better understand the intricate nature of the relationship between headache and sleep, our study focused on investigating the role and function of noradrenergic neurons in the LC during acute headache and acute sleep disturbance.

**Method:**

To explore the relationship between acute headache and acute sleep disturbance, we primarily employed nitroglycerin (NTG)-induced migraine-like headache and acute sleep deprivation (ASD) models. Initially, we conducted experiments to confirm that ASD enhances headache and that acute headache can lead to acute sleep disturbance. Subsequently, we examined the separate roles of the LC in sleep and headache. We observed the effects of drug-induced activation and inhibition and chemogenetic manipulation of LC noradrenergic neurons on ASD-induced headache facilitation and acute headache-related sleep disturbance. This approach enabled us to demonstrate the bidirectional function of LC noradrenergic neurons.

**Results:**

Our findings indicate that ASD facilitated the development of NTG-induced migraine-like headache, while acute headache affected sleep quality. Furthermore, activating the LC reduced the headache threshold and increased sleep latency, whereas inhibiting the LC had the opposite effect. Additional investigations demonstrated that activating LC noradrenergic neurons further intensified pain facilitation from ASD, while inhibiting these neurons reduced this pain facilitation. Moreover, activating LC noradrenergic neurons exacerbated the impact of acute headache on sleep quality, while inhibiting them alleviated this influence.

**Conclusion:**

The LC serves as a significant anatomical and functional region in the interaction between acute sleep disturbance and acute headache. The involvement of LC noradrenergic neurons is pivotal in facilitating headache triggered by ASD and influencing the effects of headache on sleep quality.

**Supplementary Information:**

The online version contains supplementary material available at 10.1186/s10194-024-01714-5.

## Introduction

The intricate and close relationship between sleep and headache has presented clinicians with enduring challenges for over a century [1, 2]. Headache and sleep problems are consistently encountered in clinical practice, leading to significant social and familial burdens as well as substantial socioeconomic impacts and costs [1, 3]. In 2014, a landmark epidemiological study from Denmark examined the prevalence of headache and coexisting sleep disorders and found that 18.1% of individuals reported experiencing headache and insomnia concurrently [4]. The prevalence of headache is highest among individuals aged 35 to 39, with approximately 75% of migraines commencing before the age of 35 [5, 6]. Within this age group, adults also frequently encounter acute sleep disturbance resulting from work demands, lifestyle factors, and other reasons. Consequently, it has become necessary to explore the association between acute sleep disturbance and headache.

Epidemiological studies have consistently substantiated the connection between migraine and sleep disorders [7]. Headache and sleep disturbances are widely recognized as sharing some common neurophysiological and anatomical foundations, with specific headache diagnoses closely associated with sleep disorders, a correlation rooted in the involvement of overlapping anatomical structures and neurochemical processes in the pathophysiology of both sleep and headache [1, 7–9]. Nevertheless, the relationship between headache and sleep disturbances is complex and multifaceted; establishing causal relationships between a sleep disorder and a headache in a patient can be challenging due to the usual bidirectional associations between the two [2]. The experience of pain disrupts sleep, while sleep deprivation or interference is capable of changing the perception of pain in animals and humans, potentially resulting in an intensified sensitivity to pain. Nevertheless, the exact mechanism through which sleep impacts neuropathic pain remains unclear [1, 10].

When investigating the link between sleep and headache, the focus can be split into two areas: the association between acute headache and sleep disturbances and the association between chronic headache and sleep disturbances. Moreover, these investigations can encompass research into the relationships among acute sleep disturbance, chronic sleep disturbance, and headache. The complexity of headache and sleep disturbances, combined with their intricate interplay, presents challenges in achieving a definitive solution through conventional studies. A systematic differentiation between sleep disturbances and headache across different conditions is a rational approach for gradually addressing this issue.

Therefore, we conducted a study to explore the potential relationship between sleep disorders and headache by investigating the association between acute sleep deprivation (ASD) and headache. Our previous research revealed that ASD amplifies the heightened pain sensitivity caused by nitroglycerin (NTG)-induced migraine-like headache, while the latter negatively impacts sleep quality [11]. Although we preliminarily verified these findings via mouse brain tissue under these experimental conditions, further research is still needed to explore the specific neuroanatomical regions and functional regulations involved. Hence, by employing an improved experimental design, our study seeks to investigate changes in the functional regions of the brain to uncover the potential mechanisms that underlie the interaction between ASD and headache.

## Method

All experimental procedures were approved by the Institutional Animal Care and Use Committee of the Chinese People's Liberation Army (PLA) General Hospital. Furthermore, we rigorously adhered to both the ARRIVE guidelines and the guidelines set forth by the Committee for Research and Ethical Issues of the International Association for the Study of Pain throughout all experiments [12, 13].

### Animals

Animals of both sexes ranging from 8 to 16 weeks of age were included in the experimental cohort. Specific-pathogen-free (SPF) wild-type (C57BL) mice were sourced from SiPeiFu Biotechnology Co., Ltd. (Beijing, China). DBH-Cre mice, generously provided by Professor Yulong Li at Peking University, were also incorporated into the study. The mice were housed in socially cohesive groups consisting of 4–5 animals in individually ventilated cages. Environmental conditions included a 12-hour light/dark cycle, while a standard diet was provided. To ensure unbiased distribution, the animals were randomly assigned to the different experimental conditions outlined in this investigation. Tail snips from the offspring were routinely collected and used for genotyping purposes throughout the breeding process.

### NTG-induced migraine-like headache mouse model

The NTG-induced migraine-like headache mouse model is a well-established and widely used experimental method for inducing migraine in both humans and rodents [14–16]. In systemic studies, human and animal NTG-based migraine models have greatly contributed to the identification and partial characterization of the various potential mechanisms involved in migraines [15]. Moreover, the NTG-induced migraine-like headache mouse model has particular advantages in research related to the association between sleep and headaches. The half-life of NTG is notably short, under 30 minutes [17, 18], and no study has clearly shown that NTG directly causes sleep disturbances. Considering its very short half-life, the potential of NTG to directly cause sleep disturbances is lower than that of other methods for generating headache models, such as the inflammatory soup model. As such, the NTG-induced migraine-like headache model represents an optimal headache-related model for investigating the effects of headache on sleep. Additionally, considering the variability in sleep states among individual mice, it is essential to employ suitable experimental methods that reduce the influence of these individual differences. Our earlier experiments indicated that following single NTG injections administered with a one-week gap, the pain threshold returned to normal before the subsequent injection (Supplementary Fig. 1E, F). Therefore, NTG has been shown to be particularly well suited for conducting repeated controlled experiments, enabling more accurate research into the effects of acute headache on sleep in the same mouse. Most importantly, however, NTG was employed in this study to investigate the potential interactions between migraines and sleep disorders.

NTG, purchased from Beijing Yimin Pharmaceutical Co. (Beijing, China), was prepared as a fresh dilution of 5 mg/ml in ethanol and then further diluted in 0.9% (wt/vol) saline to achieve a final concentration of 0.5 mg/ml. The administered dose was 10 mg/kg. The control group received a vehicle (VEH) solution consisting of 10% (vol/vol) alcohol, which was intraperitoneally administered at a volume of 0.2 ml/10 g. All administrations of NTG were performed intraperitoneally into the left lower abdomen. Group allocation was conducted in a double-blind manner to minimize bias. At the same time, to prevent the effects of biological rhythms on mouse pain and other behavioral results, all experiments except ASD were performed between 8 a.m. and 8 p.m. In particular, repeated experiments with the same mice were strictly limited to the same period.

### Acute sleep deprivation

In this study, we employed a widely accepted sleep deprivation model [10]. Briefly, the mice were inserted into a new automatic cylindrical device supplied by Pinnacle Technology Inc. (KS, USA). The device consists of a Plexiglas cylindrical cage, corn cob bedding and water bottles, and a rotating bar at the base. The spinning bar was calibrated at 2-4 rotations per minute over an uninterrupted period of 24 hours, from 8:00 am one day to 8:00 am the following day, to disrupt the sleep of the mice in a gentle but consistent manner. Concurrently, undisturbed mice, also housed in the same acoustic chamber, represented the control group. As previously studied and confirmed, this sleep deprivation system effectively initiates sleep deprivation in the respective subjects [19].

In this study, prior to ASD, all groups of experimental mice were rigorously maintained under identical feeding conditions, with video surveillance facilitating real-time observation. When conducting the experiment to assess the mechanical threshold changes during ASD, we abstained from performing electroencephalography (EEG) monitoring. This decision was primarily driven by the possibility of EEG placement influencing the pain domain of the mice in a way that would be challenging to quantify. To mitigate such potential impacts, we therefore refrained from performing EEG monitoring of the ASD mice.

### Cannula implantation surgery and drug delivery

The mice were anesthetized by intraperitoneal injections of 0.2 ml/10 g body weight of 1.25% avertin (Sigma, T48402, MO, USA). Subsequently, they were anesthetized with 2% isoflurane and placed in a stereotaxic instrument (RWD Life Science, Shenzhen, China). The positioning of the skull was adjusted to be on the same horizontal plane. After drilling small burr holes in the skull over the designated sites, two sets of guide cannulas were bilaterally implanted into the locus coeruleus (LC) at a 15° angle, with coordinates of -5.4 mm anterior-posterior, ±1.85 mm medial-lateral, and 3.62 mm dorsal-ventral relative to the bregma. The cannulas were then secured with anchoring screws and dental cement. To prevent clogging, a dummy cannula was inserted into each cannula. The mice were allowed a recovery period of at least one week. Prior to the induction of the NTG-induced migraine-like mouse model, the injection cannula was connected to a 10 μl microsyringe (Hamilton, Nevada, USA). The mice were then microinjected with the α2-adrenergic receptor (α2-AR) agonist clonidine (10 mg/ml in 10% DMSO in saline, B1333, APExBIO, Beijing, China) or the α2-AR antagonist yohimbine (10 mg/ml in 10% DMSO in saline, N1704, APExBIO, Beijing, China) [20–22]. The control group mice received microinjections of the same volume of vehicle in the bilateral LCs, and all mice remained awake during drug infusion.

### Surgery for virus injection, placement of EEG and electromyography (EMG) electrodes, and fiber implantation

The mice were anesthetized using Avertin and isoflurane before being placed into the same stereotaxic instrument used in the cannula implantation surgery. The skull was exposed, and its position was adjusted to align with the same horizontal plane. Small craniotomy holes were then prepared for virus injection: approximately 300 nl of AAV9-hSyn-Ne2h (Brain case, BC-0268, Wuhan, China) was microinjected into the LC (-5.4 mm anterior-posterior, ±0.95 mm medial-lateral and 3.5 mm dorsal-ventral relative to the bregma) using a fine glass pipette and a microsyringe pump (RWD Life Science, Shenzhen, China). An optical fiber cannula (Inper, Zhejiang, China) was implanted 0.1 mm above the virus injection sites to monitor the fluorescence signals and record changes in the noradrenaline concentration in the LC. AAV9-DIO-hM3Dq-mCherry, AAV9-DIO-hM4Di-mCherry or AAV9-DIO-mCherry virus (Genechem, 21514/21515/25917, Shanghai, China) was microinjected into the LC of DBH-Cre mice to carry out chemical genetic intervention.

To monitor the animal's sleep-wake state, EEG electrodes were implanted over the frontal cortex and visual cortex via craniotomy holes, while EMG wires were placed into the trapezius muscles. The electrodes were attached to a micro connector and secured to the skull using dental cement. All electrode leads were connected to a premanufactured head mount (Part #8402, Pinnacle Technology, Inc., KS, USA) and sealed with dental acrylic resin.

For fiber photometry recording and analysis, a fiber photometry system (Thinker Tech, Nanjing, China) was utilized. An optical fiber guided the light between the commutator and implanted optical fiber cannula. The excitation light power at the tip of the optical fiber was adjusted to 20–30 mW and delivered at 40 Hz to minimize photobleaching during the 6-hour recording. The green fluorescence signal was bandpass filtered and collected using a photomultiplier tube. An amplifier was employed to convert the current output from the photomultiplier tube into a voltage signal, which was then passed through a low-pass filter. The analog voltage signals were digitized using a data acquisition card (Thinker Tech, Nanjing, China). Background autofluorescence was subtracted during the subsequent analysis. The photometry data were analyzed using a custom MATLAB program. To calculate ΔF/F0, the baseline values were measured during REM sleep with no apparent fluctuations. For interanimal fluorescence comparison, the z score-transformed ΔF/F0 was further normalized using the standard deviation of the baseline signals. Simultaneously, the EEG/EMG signals were transmitted to an 8401 conditioning/acquisition system (Pinnacle Technology, KS, USA) through a tether and low-torque commutator. The 8401 amplifier/conditioning unit provided an additional 50× signal amplification, additional high-pass filtering, and an 8th-order elliptic low-pass filter (50 Hz for EEG and 200 Hz for EMG). The signals were then sampled at 1,000 ~ 2,000 Hz, digitized using an analog-to-digital converter, and routed to a PC-based acquisition and analysis software package (Pinnacle Technology, KS, USA) via USB. Subsequently, the data were scored as wake, slow-wave sleep, or REM sleep using human visual pattern recognition, and postscoring data processing was performed using the analysis package.

### Von Frey test

As previous studies had highlighted the role of the LC primarily in mechanical pain [23, 24], we prioritized analyzing changes in mechanical pain. Periorbital and hind paw mechanical allodynia were measured by using precision-calibrated Von Frey filaments (Aesthesio, CA, USA). To ensure valid test results, the mice were allowed an initial three-day period to acclimatize to their new environment. During periorbital mechanical threshold testing, the investigator cautiously maintained control of the mouse in their hand and navigated a range of standardized Von Frey filaments across the periorbital skin until the mice were noted to have a headache-like reaction. This was observed for a maximum of three seconds before determining the absence of a reaction. The presence of a headache-like reaction, indicative of a positive response, included aggressive face rubbing, head withdrawal or head shaking. Before commencing the hind paw measurements, the mice were placed in a specifically designed plexiglass case with a mesh floor. The mice were allowed to acclimate to this environment for 30 minutes. To minimize potential discomfort from intraperitoneal injections, we designated the plantar area of the right hind paw as the stimulation area. The mice were judged to respond positively to fiber stimulation until they showed signs of withdrawal or paw licking. The up-down stimulation method [25] was used, comprising five filament stimulations with the sequence and terminal filament recorded. The determination of the 50% withdrawal threshold was performed using an online algorithm freely accessible at https://bioapps.shinyapps.io/von_frey_app/  [26].

### Light/dark box test

The light/dark box (LDB) test was utilized to assess behavioral abnormalities associated with migraines. The LDB apparatus used in this study was a white polyvinyl acrylic box equipped with infrared beam tracking technology (Shanghai Xinruan Information Technology, Shanghai, China). The internal part of the LDB consisted of two sections: the box itself measured 30 cm wide × 30 cm deep × 30 cm high and was divided into two equally sized compartments. One compartment with an LED panel providing illumination of 1,000 lx served as the light chamber, while the other compartment was painted black and maintained at a light intensity of less than 5 lx to function as the dark chamber. A door positioned between the two chambers allowed the animals to freely access both compartments. To initiate the test, a mouse was placed in the dark chamber facing the wall, and its behavior was tracked for a period of 5 minutes [27].

### Immunohistochemistry

In this procedure, mice were sedated extensively with pentobarbital sodium (50 mg kg−1, i.p.) and successively perfused with 0.1 M phosphate-buffered saline (PBS, pH 7.4) and a 4% (w/v) paraformaldehyde solution. Their brains were extracted and then postfixed in a chilling environment of 4°C overnight using a 4% paraformaldehyde solution. Following postfixation, the brains were infused sequentially with 15% (wt/vol) and 30% (wt/vol) sucrose solutions until they were submerged at the bottom of the solvent. Thereafter, the brains were embedded in O.C.T. compound and sectioned into 30-μm coronal slices employing a freezing microtome (Leica Biosystems, CM1950, Heidelberger, Germany). The brain sections were incubated in blocking buffer consisting of 10% (vol/vol) normal donkey serum and 0.25% (vol/vol) Triton X-100 diffused in PBS for 2 hours at room temperature. Subsequently, they were incubated with primary antibodies, including anti-c-Fos (1:1,000, guinea pig, SYSY, Gottingen, Germany) and anti-dopamine beta hydroxylase (1:1,000, ab209487, rabbit, Abcam, MA, USA), for 12 hours at 4°C, followed by exposure to the corresponding fluorophore-conjugated secondary antibodies for 2 hours at room temperature. After three sequential washes with PBS plus Tween (PBST), each lasting 30 minutes, the sections were mounted and coverslipped with DAPI mounting medium (Abcam, ab104139, MA, USA), and the fluorescence signals were visualized using an Olympus VS120 (Olympus, Tokyo, Japan).

### Quantification and statistical analysis

In these experiments, the mice were randomly divided into several groups according to a benchmark derived from past experience and research. The acute brain slices of the mice served as the source of our images, which were subsequently processed via ImageJ software (Fiji, NIH, USA). Additionally, the software was used to subtract the background levels measured outside the region of interest in the images. Statistical analyses were performed using Prism 8 (GraphPad, CA, USA). Unless stated otherwise, all data are summarized as the mean ± standard error of the mean (S.E.M.). The paired or unpaired Student's t test was used to compare two groups, while for comparisons involving more than two groups, either one-way ANOVA, two-way ANOVA, or a two-way repeated-measures ANOVA was chosen followed by Tukey's post hoc test. All statistical tests were two-tailed, and *P <*0.05 was recognized as indicating statistical significance.

## Results

### ASD exacerbates acute NTG-induced hyperalgesia

In our previous studies, we showed that ASD can facilitate the sensitization of pain in response to NTG. Expanding on these findings, we employed a low-speed rotating rod method to induce ASD in this study. Through this protocol, we provided additional evidence for the facilitation of NTG-induced headache by ASD.

**Fig. 1:**
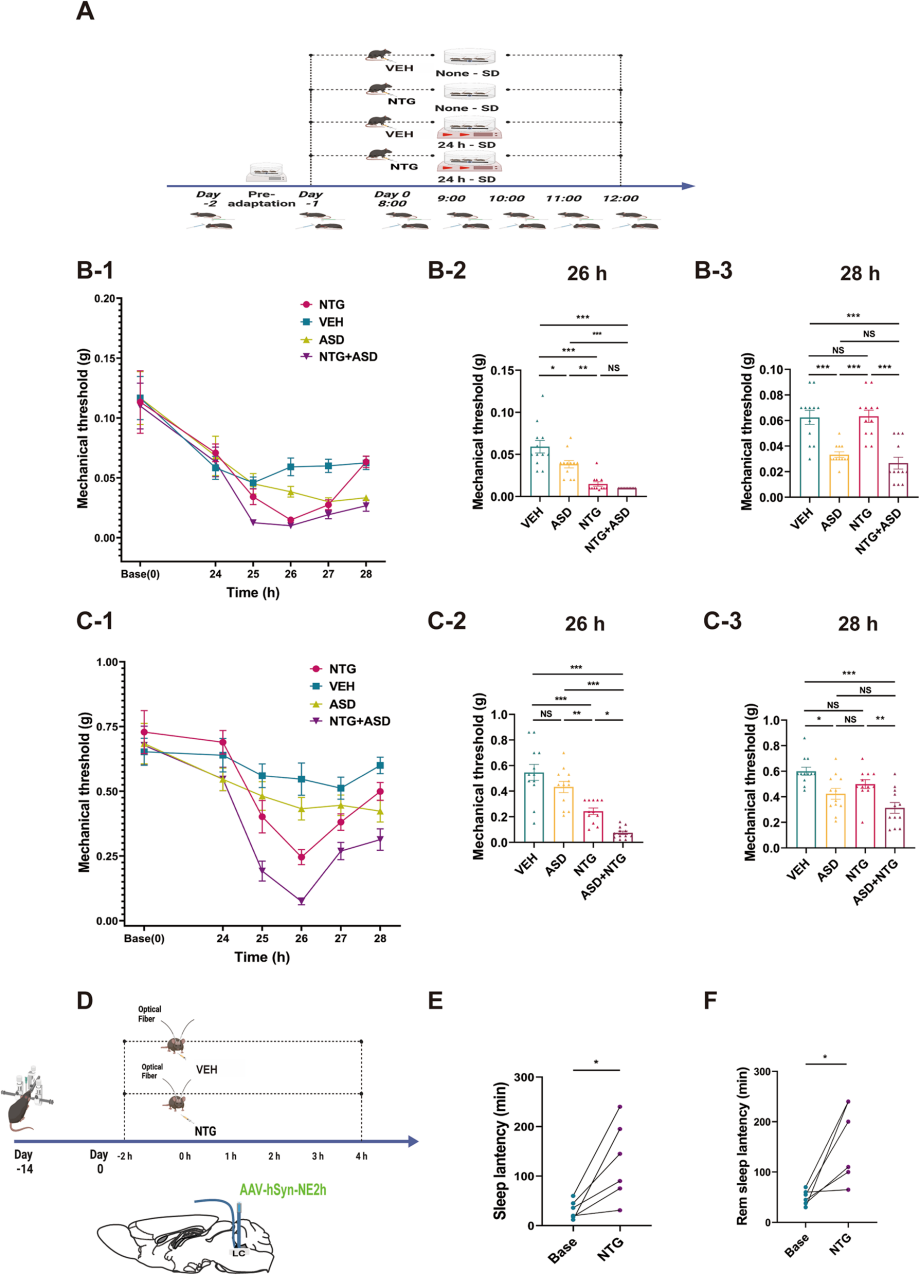
ASD intensifies the hyperalgesia evoked by NTG, and acute administration of NTG promotes sleep disturbances. **A** Schematic depiction of the timeline demonstrating the aggravating influence of ASD on NTG-induced hyperalgesia (VEH, *n =* 12 mice; ASD, *n =* 12 mice; NTG, *n =* 12 mice; NTG+ASD, *n =* 12 mice). **B** and **C** Temporal dynamics of the changes in the periorbital mechanical threshold (B-1) and right hind paw (**C**-**1**) induced by NTG, as determined by von Frey tests. Furthermore, the graphical representations in **B**-**2**/**C**-**2** demonstrate statistical trends recorded at the 26-hour time point within the timeline (1.5 to 2 hours post NTG injection), and **B**-**3**/**C**-**3** demonstrate statistical trends recorded at the 28-hour time point within the timeline. **D** Schematic depiction of the timeline showing the acute administration of NTG leading to sleep disturbances. **E** and **F** The acute administration of NTG impacts both sleep latency and sleep duration (*n =* 6). Data are expressed as the mean ± SEM. Two-way ANOVA with Tukey's post hoc test for multiple comparisons and paired Student's t test for two groups. * *P <* 0.05; ** *P <* 0.01; ***P < 0.001. (**B**-**2**: *F =* 24.96, VEH vs. ASD *P* = 0.014, vs. NTG *P <* 0.001, vs. NTG + ASD *P <* 0.001; ASD vs. NTG *P* = 0.005, vs. NTG+ASD *P <* 0.001; NTG vs. NTG+ASD *P* = 0.875. **B**-**3**: *F =* 19.99, VEH vs. ASD *P <* 0.001, vs. NTG *P >* 0.9, vs. NTG + ASD *P <* 0.001; ASD vs. NTG *P <* 0.001, vs. NTG+ASD *P <* 0.001; NTG vs. NTG+ASD *P* = 0.875. *F =* 24.96, VEH vs. ASD *P* = 0.014, vs. NTG *P <* 0.001, vs. NTG + ASD *P >* 0.9; ASD vs. NTG *P <* 0.001, vs. NTG+ASD *P >* 0.9; NTG vs. NTG+ASD *P <* 0.001. **C**-**2**: *F* = 25.44, VEH vs. ASD *p* = 0.224, vs. NTG *p <* 0.001, vs. NTG+ASD *p <* 0.001; ASD vs. NTG *p* = 0.015, vs. NTG+ASD *p <* 0.001; NTG vs. NTG+ASD p = 0.029. **C**-**3**: *F =* 10.91, VEH vs. ASD *P* = 0.009 vs. NTG *P* = 0.230, vs. NTG+ASD *P <* 0.001; ASD vs. NTG *P* = 0.471, vs. NTG+ASD *P* = 0.167; NTG vs. NTG+ASD *P* = 0.006. **E**: t = 3.368, Base vs. NTG *P* = 0.020. **F**: t = 3.445, Base vs. NTG *P* = 0.018)

In this study (Fig. 1A), baseline periorbital and hind paw mechanical pain thresholds were measured before the initiation of ASD. At 24 hours after the baseline pain threshold measurements, each of the four groups received either NTG or VEH. Subsequently, the mechanical pain threshold was assessed at hourly intervals for a duration of 4 hours following NTG or VEH injection (Fig. 1A). In addition, in another set of experiments, we tested the behavior in the LDB 1.5 to 2 hours after NTG or VEH injection (Supplementary Fig. 1A).

**Fig. 2:**
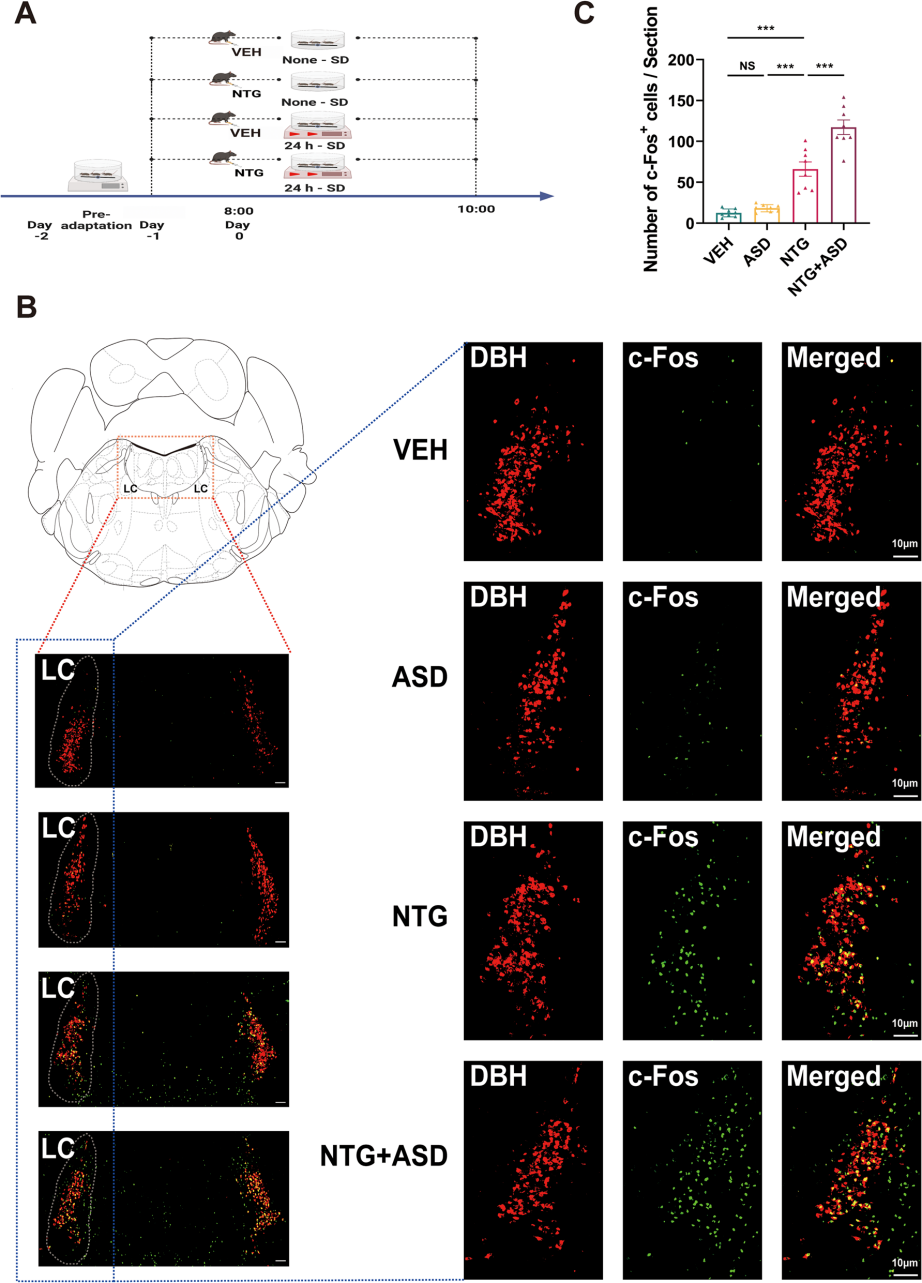
Activation of noradrenergic neurons in the LC potentially contributes to the influence of ASD on NTG-induced hyperalgesia (VEH, *n =* 8 mice; ASD, *n =* 8 mice; NTG, *n =* 8 mice; NTG+ASD, *n =* 8 mice). **A** Schematic representation of the experimental timeline and the designated sampling time for evaluating the mouse brain. **B** Representative LC images obtained from the VEH, ASD, NTG, and NTG+ASD mouse brain groups. For each group, the left panel shows an overview of the region (scale bar, 100 μm), while the right panels show enlargements of the region of interest (scale bar, 10 μm). The white contour precisely delineates the borders of the LC. **C** Graphs for c-Fos+ cell counts in the LC (*n =* 8, each group). One-way ANOVA with Tukey's post hoc test for multiple comparisons. *P < 0.05, **P < 0.01 and ***P < 0.001 vs. the indicated group. (C: VEH vs. ASD *P* = 0.918, vs. NTG *P <* 0.001, vs. NTG+ASD *P <* 0.001; ASD vs. NTG *P <* 0.001, vs. NTG+ASD *P <* 0.001; NTG vs. NTG+ASD *P <* 0.001)

**Fig. 3:**
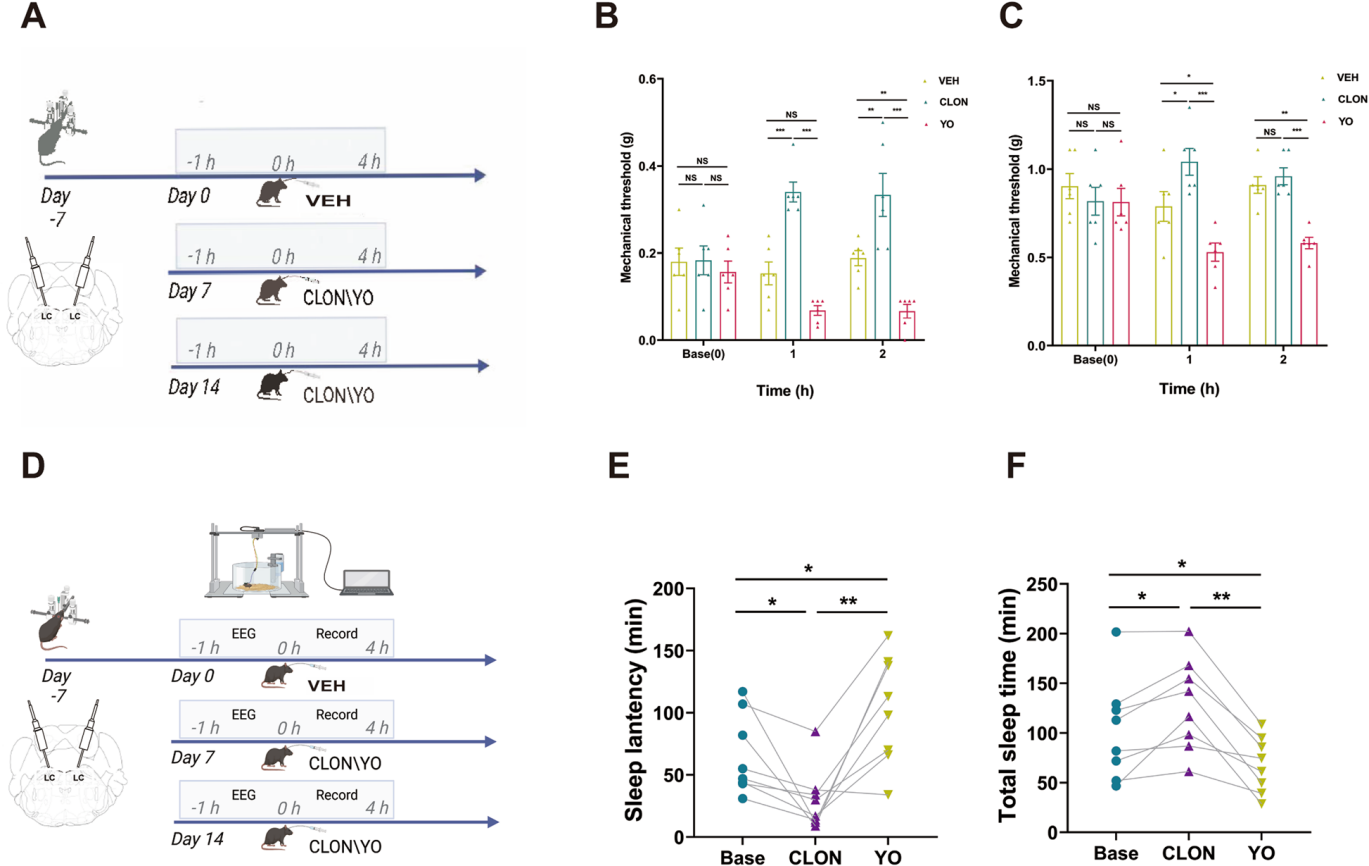
Administration of α2 receptor agonists/antagonists through microinjection in the LC can modify pain perception and sleep architecture. **A** Schematic representation of the timeline demonstrating the change in mechanical pain triggered by agonists/antagonists, as measured via von Frey tests (*n =* 6 mice). **B**/**C** Temporal dynamics of changes and statistical trends in the periorbital mechanical threshold (**B**) and right hind paw threshold (**C**) induced by agonists/antagonists. **D** Schematic representation of the timeline demonstrating the change in sleep architecture triggered by agonists/antagonists (*n =* 6 mice). **E/F** Statistical trends in sleep latency (**E**) and sleep time (**F**) induced by agonists/antagonists. Data are expressed as the mean ± SEM. Two-way ANOVA with Tukey's post hoc test for multiple comparisons and one-way repeated-measures ANOVA for multiple comparisons. * *P <* 0.05; ** *P <* 0.01; ***P < 0.001. (**B**: Base CLON vs. VEH *P* = 0.998, vs. YO *P* = 0.778, VEH vs. YO *P* = 0.825; **1h**: Base CLON vs. VEH *P <* 0.001, vs. YO *P <* 0.001, VEH vs. YO *P* = 0.090; **2h**: Base CLON vs. VEH *P* = 0.002, vs. YO *P <* 0.001, VEH vs. YO *P* = 0.009.**C**: Base CLON vs. VEH *P* = 0.631, vs. YO *P* = 0.998, VEH vs. YO *P* = 0.598; **1h**: Base CLON vs. VEH *P* = 0.024, vs. YO *P <* 0.001, VEH vs. YO *P* = 0.021; **2h**: Base CLON vs. VEH *P* = 0.852, vs. YO *P <* 0.001, VEH vs. YO *P* = 0.003.**E**: Base vs. CLON *P* = 0.049, vs. YO *P* = 0.029; CLON vs. YO *P* = 0.006. **F**: Base vs. CLON *P* = 0.035, vs. YO *P* = 0.045; CLON vs. YO *P* = 0.003)

Following the 24-hour ASD procedure, all four groups exhibited a gradual decline in the pain threshold (Fig. 1B-1, C-1). There were no statistically significant differences in the mechanical pain threshold between the periorbital and hind paw regions among the groups at 24 hours. At 26 hours, two hours after NTG administration, both the periorbital and hind paw pain thresholds reached their nadirs (Fig. 1B-1, C-1). At this point, no significant differences were observed in the mechanical pain domains between the normal control group and the ASD group, and the periorbital mechanical pain threshold was significantly lower in the ASD group than in the control group (Fig. 1B-2, C-2). However, a dissimilarity in hind paw pain but not periorbital pain was evident between the NTG+ASD and NTG groups. Our findings indicated that ASD potentiated NTG-induced pain, which was further demonstrated by the results of the LDB behavioral test conducted at 26 hours, which revealed differences between the ASD+NTG and NTG groups. During the subsequent recovery phase (between 26 and 28 hours) (Fig. 1B-1, C-1), the pain threshold of the ASD+NTG group remained considerably different from that of the NTG group at the 4-hour interval after NTG or VEH injection (Fig. 1C-3), suggesting that ASD prolonged the duration of NTG-induced headache.

Additionally, we observed a gradual decrease in the pain threshold of ASD mice during the 1-hour pain zone measurement interval (Fig. 1B-1, C-1), which could be attributed to potential alterations in the behavior of the mice toward the number of mechanical pain zone tests following long-term ASD. Thus, in subsequent experiments, we reduced the frequency of mechanical pain measurements during the ASD state and carried out only two pain threshold tests, one before the injection and the other 1.5–2 hours after the injection. The purpose of this modification was to minimize potential confounding factors that could influence the assessment of the mechanical pain field.

We also conducted experiments on the effect of NTG-induced migraine-like headache on sleep. In the first experiment, we introduced a fluorescent probe via virus injection (Fig. 1D) into the LC region, allowing us to accurately assess the impact of NTG on sleep patterns through fluorescence monitoring [28]. Fluorescent probe technology can reflect changes in noradrenaline concentration, which is closely related to sleep structure and can reflect the sleep state [28, 29]. Subsequent experiments successfully corroborated a clear relationship between changes in fluorescence patterns and alterations in sleep structure (Fig. 6B). Notably, the findings from this study conclusively demonstrated that NTG administration leads to an increase in sleep latency and particularly REM sleep latency (Fig. 1E, F). Our previous investigations also previously indicated that NTG-induced migraine-like headache can potentially increase the sleep onset time and disrupt sleep structure [11].

Indeed, during the experimental process, we discovered that the significant changes in the mechanical pain field around the mouse's periorbital area were not in full accordance with those in the hind paw mechanical pain field. This was partly due to the lower threshold of the periorbital mechanical pain, resulting in smaller changes. On the other hand, when measuring the periorbital mechanical pain, the mouse's pain expression varied and was more challenging to judge. We conducted three preadaptations before making the official measurements. However, after the onset of ASD, all four groups showed a downward trend in the orbital pain field. Moreover, further measurements could not be made in a portion of the pain field in the ASD+NTG group at the lower limit of 0.008 g. Concurrently, the chemogenetic results also reflected this characteristic change in the periorbital pain field (Fig. 5 B and D).  We are inclined to believe that the measurement of the periorbital pain area is more susceptible to interference, which led to the instability of the results. In contrast, the plantar pain field yielded a more objective and stable performance. Nevertheless, from the trends of pain field changes and the statistical perspective, the changes in the periorbital pain field and plantar pain field were essentially consistent. Therefore, our conclusions are primarily based on the changes in the plantar pain field, with the periorbital pain field serving as an important reference.

In summary, the present study demonstrates that ASD amplifies hyperalgesia in NTG-induced migraine-like headache and prolongs its duration, while NTG-induced migraine-like headache disrupts sleep structure.

### Alterations in noradrenergic neurons within the LC are linked to the interplay between ASD and NTG-induced migraine-like headache

To further elucidate the brain regions implicated in the interaction between acute pain and ASD, we conducted an experiment in which brain tissue sections were prepared and subjected to fluorescence staining during NTG-induced migraine-like headache through ASD.

The design of the experiments conducted in this particular set paralleled the strategies employed in the preceding groups, which involved the random assignment of the mice into four distinct groups (Fig. 2A). By analyzing the trends in pain threshold changes (Fig. 1B1/C1) and drawing upon our previous studies, we ascertained that NTG-induced migraine-like headache achieved its peak reduction at 1.5–2 hours after NTG injection. Considering the expression characteristics of c-Fos, the mice were anesthetized 2 hours after NTG injection ,then the brain tissue samples were taken for processing. After the staining procedure, we noted a consistent pattern in the changes in the amount of c-Fos-positive neurons within the LC region that corresponded to the alterations observed during earlier experiments (Fig. 2C). To further substantiate our findings, we examined the specific cell types within the LC region and intriguingly observed a strong correlation between noradrenergic and c-Fos-positive neurons by double immunofluorescence staining  (Fig. 2B).

Therefore, based on the evidence at hand, we tentatively posit that LC noradrenergic neurons may be implicated in the interplay between acute sleep disturbance and acute headache.

### LC noradrenergic neurons are implicated in both sleep quality and pain regulation

To assess the potential involvement of LC noradrenergic neurons in the interplay between sleep and headache, we conducted an initial investigation into their contributions to the pain and sleep processes using localized drug administration (Fig. 3A, D). Prior studies have indicated that neurons in the LC region predominantly express α2-AR and can be modulated by α2-AR agonists or antagonists [30]. Consequently, we selectively triggered or inhibited these noradrenergic neurons in the LC through microinjections of antagonists (yohimbine, promoting noradrenergic neurons) and agonists (clonidine, suppressing noradrenergic neurons), respectively. Our results indicated that the drug-induced activation of LC noradrenergic neurons led to a reduction in the pain threshold (Fig. 3B, C) and sleep duration (Fig. 3F). Conversely, inhibition of these neurons resulted in an increase in the pain threshold (Fig. 3B), an increase in sleep duration (Fig. 3F) and a decrease in sleep latency (Fig. 3E).

Correspondingly, chemogenetic intervention statistically demonstrated that noradrenergic neuron activation led to a reduction in the hind paw pain threshold between the clozapine N-oxide (CNO) group and VEH group, and the periorbital mechanical threshold showed a downward trend (Fig. 5B). Furthermore, inhibition of noradrenergic neurons resulted in an increase in the periorbital mechanical threshold between the CNO group and VEH group, and the hind paw pain threshold showed an upward trend (Fig. 5D). In fact, there was a consistent trend in some outcomes between the pharmacological and chemogenetic interventions, but there was no statistically significant difference in other outcomes. With the two interventions complementing each other, the overall result is sufficient.

### Intervention of noradrenergic neurons in the LC alters the course of NTG-induced acute migraine and of the ASD facilitation of mechanical pain perception

**Fig. 4:**
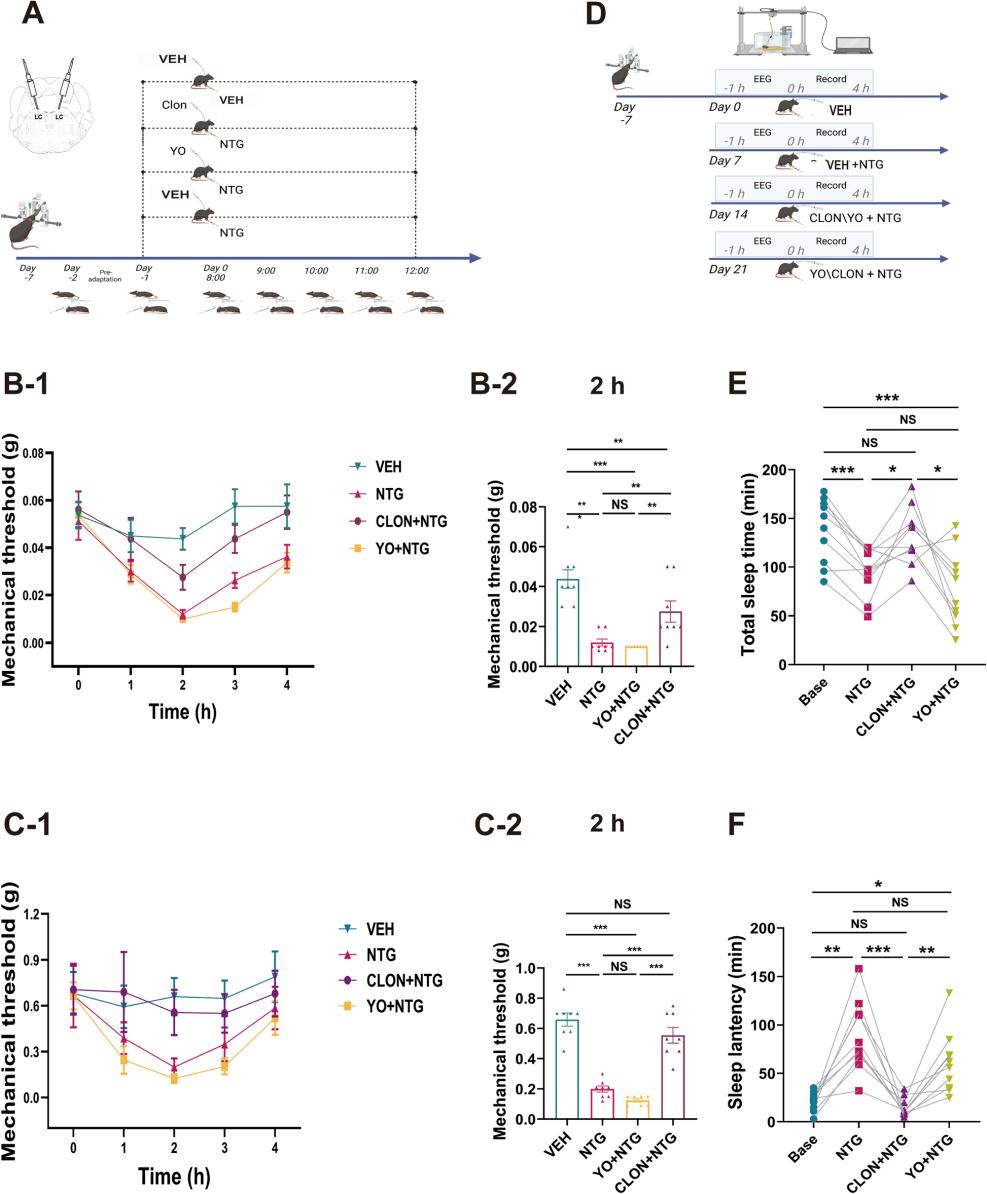
Administration of α2 receptor agonists/antagonists through microinjection in the LC affects the interaction between sleep and acute pain (VEH, *n =* 8 mice; NTG, *n =* 8 mice; NTG+CLON, *n =* 8 mice; NTG+YO, *n =* 8 mice). **A** Schematic representation of the temporal sequence illustrating the modification in NTG-induced hyperalgesia, as measured through von Frey tests, after the administration of agonists or antagonists. **B** and **C** Temporal dynamics of the changes in the periorbital mechanical threshold (**B**-**1**) and right hind paw (**C**-**1**) induced by NTG and agonists/antagonists, as determined by von Frey tests. Graphical representations of **B**-**2**/**C**-**2** demonstrated statistical trends recorded at the 26-hour time point within the timeline (1.5 to 2 hours post NTG injection). **D** Schematic depiction of the timeline showing the acute administration of NTG and agonists/antagonists leading to sleep disturbances (*n =* 10 mice). **E** and **F** The acute administration of NTG leads to significant alterations in both sleep latency and sleep duration, with this change being regulated by the microinjection of α2 receptor agonists and antagonists. Data are expressed as the mean ± SEM. Two-way ANOVA with Tukey's post hoc test for multiple comparisons and one-way repeated-measures ANOVA for multiple comparisons. * *P <* 0.05; ** *P <* 0.01; ***P < 0.001. (**B-2**: VEH vs. NTG *P <* 0.001, vs. CLON+NTG *P* = 0.006, vs. YO+NTG *P <* 0.001; NTG vs. CLON+NTG *P* = 0.009, vs. YO+NTG *P* = 0.967; CLON+NTG vs. YO+NTG *P* = 0.003. **C-2**: VEH vs. NTG *P <* 0.001, vs. CLON+NTG *P* = 0.203, vs. YO+NTG *P <* 0.001; NTG vs. CLON+NTG *P <* 0.001, vs. YO+NTG *P* = 0.467; CLON+NTG vs. YO+NTG *P <* 0.001. **E**: Base vs. NTG *P <* 0.001, vs. CLON+NTG *P* = 0.965, vs. YO+NTG *P* = 0.002; NTG vs. CLON+NTG *P* = 0.035, vs. YO+NTG *P* = 0.524; CLON+NTG vs. YO+NTG *P* = 0.040. **F**: Base vs. NTG *P* = 0.002, vs. CLON+NTG *P* = 0.604, vs. YO+NTG *P* = 0.020; NTG vs. CLON+NTG *P* = 0.001, vs. YO+NTG *P* = 0.507; CLON+NTG vs. YO+NTG *P* = 0.006)

To further elucidate the involvement of LC noradrenergic neurons in acute sleep disturbance and acute headache, we first conducted investigations into their ability to modulate the pain process induced by NTG through local drug administration (Fig. 4A). The activation of noradrenergic neurons through drug administration during the NTG-induced pain process did not lead to a further reduction in the pain threshold (Fig. 4B-1, B-2, C-1, C-2). Conversely, inhibiting noradrenergic neurons significantly alleviated the reduction in the NTG-induced pain threshold.

To study the potential involvement of LC noradrenergic neurons in the bidirectional relationship between acute sleep disturbance and acute headache, we employed chemogenetic methods to selectively modulate the activity of LC noradrenergic neurons and observe their impact on the ASD-induced pain modulation process.

**Fig. 5:**
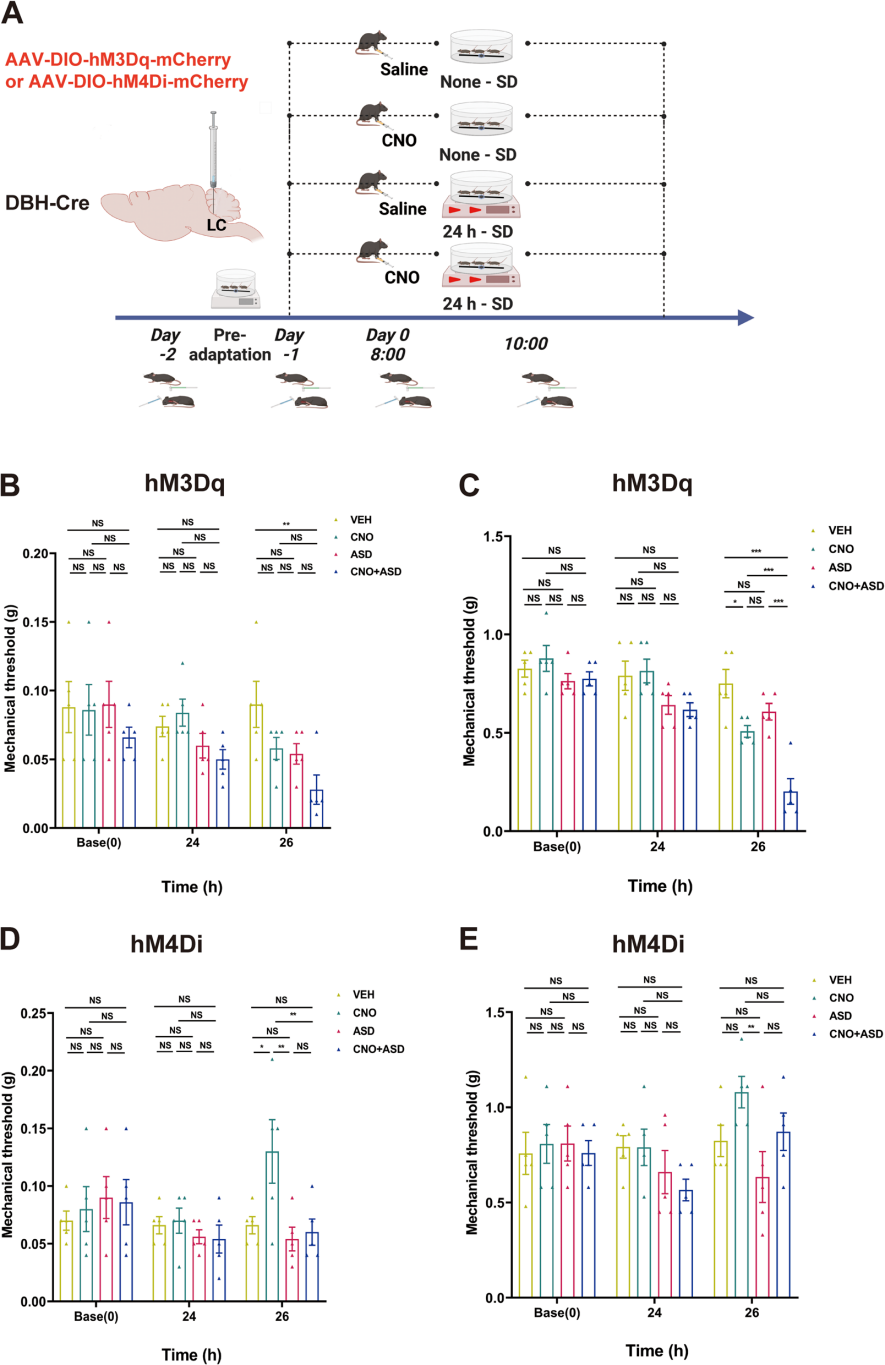
Chemogenetic modulation of noradrenergic neuron activity in the LC significantly influences the impact of sleep deprivation on pain perception (VEH, *n =* 5 mice; CNO, *n =* 5 mice; ASD, *n =* 5 mice; CNO+ASD, *n =* 5 mice). **A** Schematic representation of the timeline elucidating the modification of the mechanical pain response induced by ASD and subsequent intervention employing chemogenetic inhibition and activation. **B** and **C** Temporal dynamics of changes and statistical trends in the periorbital mechanical threshold (**B**) and right hind paw threshold (**C**) induced by chemogenetic activation. **D** and **E** Temporal dynamics of changes and statistical trends in the periorbital mechanical threshold (**D**) and right hind paw threshold (**E**) induced by chemogenetic inhibition. Data are expressed as the mean ± SEM. Two-way ANOVA with Tukey's post hoc test for multiple comparisons. * *P <* 0.05; ** *P <* 0.01; ***P < 0.001. (**B**: **26h**: VEH vs. CNO+ASD *P* = 0.005. There was no significant difference in the comparative weight of other groups at baseline, 24h and 26h. **C**: VEH vs. CNO *P* = 0.012, vs. ASD *P* = 0.244, vs. CNO+ASD *P <* 0.001; CNO vs. ASD P= 0.546, vs. CNO+ASD *P <* 0.001; ASD vs. CNO+ASD *P <* 0.001. There was no significant difference in the comparative weight of other groups at baseline and 24h. **D**: VEH vs. CNO *P* = 0.017, vs. ASD *P* = 0.938, vs. CNO+ASD *P* = 0.991; CNO vs. ASD P= 0.003, vs. CNO+ASD *P* = 0.008; ASD vs. CNO+ASD *P* = 0.991. There was no significant difference in the comparative weight of other groups at baseline and 24h. **E**: 26h: CNO vs. ASD *P*=0.008. There was no significant difference in the comparative weight of other groups at baseline, 24h and 26h)

We observed that activation of noradrenergic neurons resulted in a decrease in the pain threshold (Fig. 5B). Moreover, ASD facilitated the pain threshold reduction caused by the activation of noradrenergic neurons (Fig. 5B, C). Conversely, inhibition of noradrenergic neurons led to an improvement in the periorbital pain threshold (Fig. 5D) but had no significant influence on the hind paw pain threshold (Fig. 5E). However, the analgesic effect of noradrenergic neuron inhibition differed significantly from the facilitation effect induced by ASD (Fig. 5D, E).

In conclusion, noradrenergic neurons in the LC region are substantially involved in the NTG-induced pain mechanism as well as in the ASD facilitation of mechanical pain.

### The modulation of noradrenergic neurons within the LC influences the exacerbation of sleep disturbances during acute NTG-induced migraine

Furthermore, we employed chemogenetic techniques to specifically manipulate noradrenergic neurons to investigate whether such manipulations could alter the pain-sleep relationship.

**Fig. 6:**
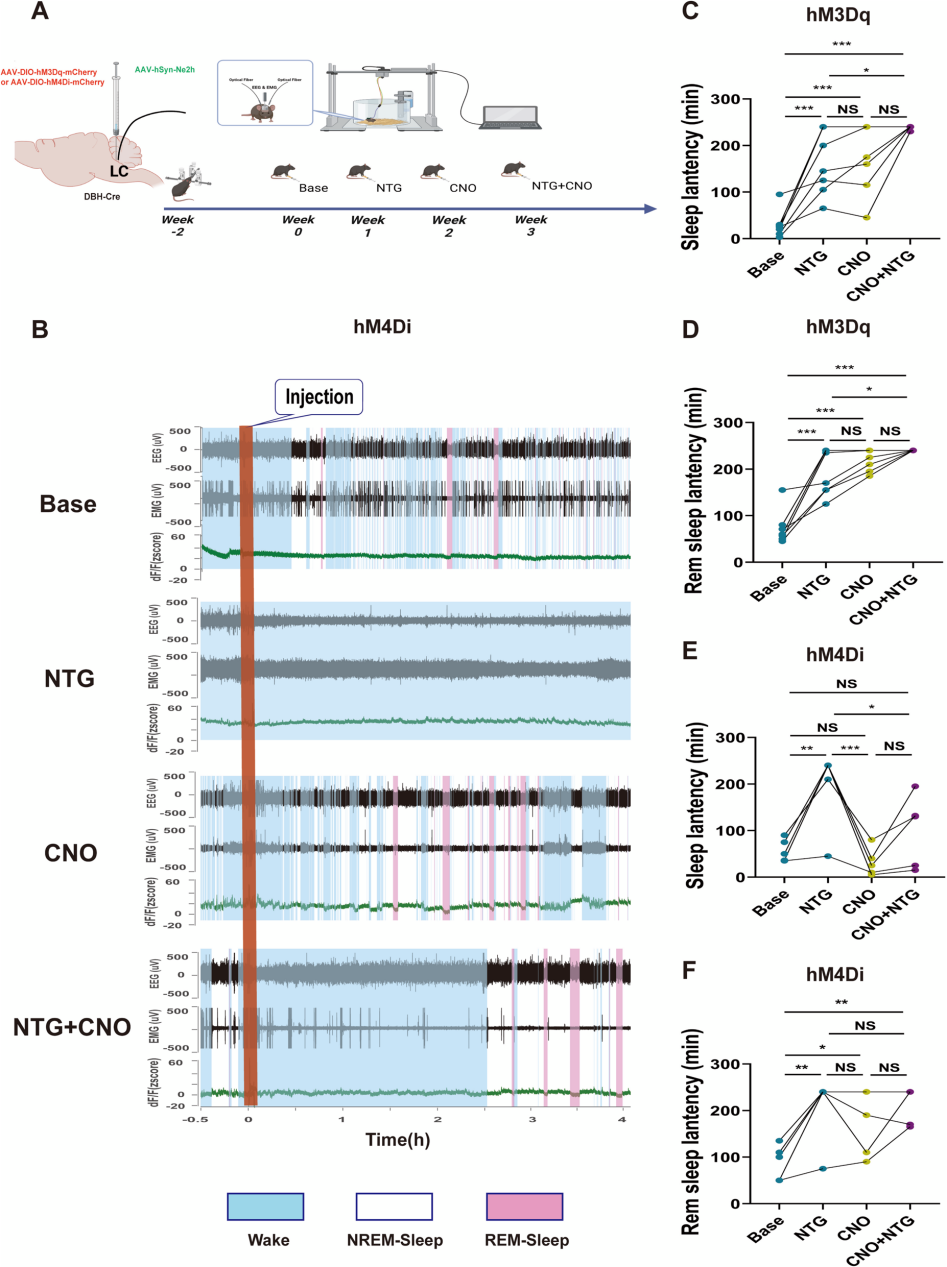
Chemogenetic modulation of noradrenergic neuron activity in the LC significantly influences the impact of acute pain on sleep architecture (*n*=6). **A** Schematic representation of the timeline depicting the acute administration of NTG and the utilization of chemogenetic techniques, leading to modifications in sleep architecture. **B** A representative image illustrating simultaneous recordings of EEG, EMG, and neuronal activity (Ne2h) in a DBH-Cre mouse highlights the significant influence of CNO on AAV-DIO-hM4Di-mCherry noradrenergic neurons, revealing its impact on the modulation of sleep architecture in response to acute pain. **C** and **D** Statistical trends of the effects of the activation of noradrenergic neurons on sleep architecture in response to acute pain. **E** and **F** Statistical trends of the effects of the inhibition of noradrenergic neurons on sleep architecture in response to acute pain. Data are expressed as the mean ± SEM. Two-way ANOVA with repeated measures for multiple comparisons. * *P <* 0.05; ** *P <* 0.01; ***P < 0.001. (**C**: Base vs. NTG *P <* 0.001, vs. CNO *P <* 0.001, vs. CNO+NTG *P <* 0.001; NTG vs. CNO *P* = 0.944, vs. CNO+NTG *P* = 0.022; CNO vs. CNO+NTG *P* = 0.069. **D**: Base vs. NTG *P <* 0.001, vs. CNO *P <* 0.001, vs. CNO+NTG *P <* 0.001; NTG vs. CNO *P* = 0.264, vs. CNO+NTG *P* = 0.025; CNO vs. CNO+NTG *P* = 0.587. **E**: Base vs. NTG *P* = 0.003, vs. CNO *P* = 0.839, vs. CNO+NTG *P* = 0.529; NTG vs. CNO *P <* 0.001, vs. CNO+NTG *P* = 0.037; CNO vs. CNO+NTG *P* = 0.173. **F**: Base vs. NTG *P* = 0.002, vs. CNO *P* = 0.021, vs. CNO+NTG *P* = 0.002; NTG vs. CNO *P* = 0.551, vs. CNO+NTG *P* = 0.998; CNO vs. CNO+NTG *P* = 0.459)

We discovered that activation of LC noradrenergic neurons could independently increase sleep latency (Fig. 6C, D), which was completely consistent with the results from drug intervention (Fig. 3E). Notably, in combination with NTG, noradrenergic neuron activation significantly enhanced the impact of NTG on sleep disturbance (Fig. 6C, D), which was also consistent with the results from the drug intervention (Fig. 4D, E, F). Conversely, the inhibition of LC noradrenergic neurons did not exert a significant effect on sleep latency alone (Fig. 6E), but when this inhibition was paired with NTG, it effectively inhibited the influence of the latter on sleep (Fig. 6F). For the corresponding drug experiments, the drug interventions shown in Fig. 4E and F also resulted in significant changes.

From the above results, we can conclude that acute NTG-induced migraine-like headache can lead to acute sleep disturbance, and changes in LC noradrenergic neurons play an important role in this process. However, in the experiment in which ASD led to increased susceptibility to headache, although LC noradrenergic neurons were not significantly activated by ASD, their reactivity to NTG was enhanced by ASD, and they were more easily activated. In conclusion, both the drug-based and chemogenetic investigations demonstrated that alterations in LC noradrenergic neuron activity can significantly impact the facilitation of pain induced by ASD as well as the influence of pain on acute sleep disturbance. This highlights the crucial role of LC noradrenergic neurons as essential anatomical components involved in the interaction between acute sleep disturbance and acute headache.

## Discussion

Our experiments found that LC noradrenergic neurons have an important function in the interaction between ASD and NTG-induced migraine-like headache. This observation supports the hypotheses posited in a earlier literature review in which the LC acts as a shared pathway linking pain and sleep, thus potentially assuming a critical role in the reciprocal relationship between the two [7–9, 31–38].

In our investigation, we observed that NTG-induced migraine-like headache precipitated acute sleep disturbance, while similarly, ASD heightened the susceptibility to headache. This suggests a bidirectional relationship wherein a specific anatomical structure may be influenced by, and in turn influence, two distinct states. Importantly, these states appear to superimpose rather than negate each other. Consequently, shared neuroanatomical structures may underlie both headache and acute sleep disturbance. The intersection between the sleep and headache pathways manifests predominantly within the diencephalic and brainstem structures of the brain, comprising the LC [2]. These neural structures send monoaminergic projections to the thalamic nuclei, lateral hypothalamus, basal forebrain and cortex, playing crucial roles in regulating arousal levels and exhibiting heightened activity during wakefulness [39]. Beyond their contribution to wakefulness, these brainstem structures, including the LC, also assume an important function in modulating the perception of headache [2, 5]. To investigate the effects of short-term ASD and its interaction with pain facilitation, we employed a well-established experimental design. First, we developed a migraine-like headache model induced by NTG. Subsequently, c-Fos staining was employed to label potentially relevant brain regions, including the LC and periaqueductal gray, within this model. Notably, the mechanical pain responses in the headache-model mice consistently demonstrated a similar trend in the paw and periorbital areas (Fig. 1B, C). These findings indicate that noradrenergic neurons were not significantly activated by short-term ASD alone. However, they exhibited heightened sensitivity to activation, potentially playing a substantial role in ASD-induced headache facilitation.

To further explore the relationship between LC and ASD and headache, we divided our experiment in two. First, we confirmed the involvement of LC cells in the process of ASD-induced headache facilitation. Next, we also confirmed the participation of LC cells in the process of NTG affecting sleep. For each experiment, we conducted essential verification steps to separately validate the role of LC noradrenergic neurons in headache and sleep. The involvement of LC noradrenergic neurons in these processes has been consistently demonstrated in previous studies [9, 38, 40]. When examining the role of LC noradrenergic neurons in ASD-induced pain facilitation, we employed both pharmacological and chemical genetic approaches to confirm their contributions and found that the LC was an important mediator rather than an antagonist of this process. Similarly, in our investigation of the effects of NTG on sleep, we utilized drug and chemical genetics to modulate the function of LC noradrenergic neurons, providing conclusive evidence for the crucial functional role of the LC in the interplay between headache and sleep.

The results obtained from both our drug experiments and our chemical experiments consistently demonstrated the same trends. Following the chemogenetic inhibition of LC noradrenergic neurons alone, there was no significant enhancement observed in the mechanical pain threshold. However, upon inducing headache with NTG, the chemogenetic inhibition of LC noradrenergic neurons led to a notable increase in the pain threshold. Concurrently, to ascertain whether drug stimulation and suppression directly influence alterations in the pain domain, we established groups for drug stimulation/suppression interventions (Fig. 3A). The findings suggest that the pure stimulation of noradrenergic neurons could diminish the pain domain, while suppressing noradrenergic neurons can enhance the pain domain. It is important to consider that the measurement process of the mechanical pain threshold itself may be influenced by factors such as the level of consciousness or the presence of nonnoradrenergic neurons in the LC in the mice. Furthermore, the administration of clonidine/yohimbine may have varying effects on the consciousness level or on nonnoradrenergic LC neurons of the mice. This can explain the further improvement in pain after clonidine inhibited the noradrenergic neurons, building upon the existing baseline threshold. Therefore, a more conclusive statement can be made: the activation of LC noradrenergic neurons can induce pain, while their inhibition can ameliorate the decline in the pain threshold caused by headache.

Throughout the process of headache-related sleep disruption, activation of LC noradrenergic neurons results in an augmented manifestation of sleep disorders, while inhibition counteracts the severity of these disorders. In this study, we incorporated sleep monitoring in combination with a noradrenergic probe to accurately gauge the sleep patterns of the mice. Notably, we observed a significant increase in brain electrical activity and a reduction in muscle activity among mice experiencing pain (Fig. 6B), thereby exerting an influence on their sleep architecture. By leveraging fluorescent probing, we were able to more precisely assess the sleep state of the mice, thereby bolstering the reliability of our experimental findings.

Consistent with these findings, previous studies have also demonstrated that neuropathic pain leads to sleep disturbances in mice and rats [41, 42]. Additionally, studies have observed heightened activity of LC-prefrontal cortex (PFC) noradrenergic neurons in mice [42] and found that chemogenetic activation of these neurons exacerbated spontaneous foot-lifts in rats with tibial nerve injury [42]. These observations suggest that overactivation of the LC induced by neuropathic pain may contribute, at least partially, to the sleep disturbances associated with chronic pain [38].

However, it is worth noting that there are some inconsistencies between our experimental findings and previous studies. Prior research has indicated that the LC primarily functions as an endogenous "antinociceptive system" [2, 43], and clonidine has a good therapeutic effect on some headache patients [44, 45]. Specifically, clonidine has been shown to inhibit the LC and have an ameliorating effect on pain perception. In previous literature, the analgesic effect of clonidine was primarily attributed to the activation of α2-AR in the spinal cord. In contrast, our experiments suggest that the analgesic effect of clonidine may not solely rely on the inhibition of spinal cord receptors but rather on the direct inhibition of LC noradrenergic neurons via microinjection at the site of the LC, leading to a decrease in terminal noradrenergic release. Consequently, the injection of clonidine should result in reduced pain perception. However, contrary to the hypothesis that clonidine alleviates pain, it should be acknowledged that leakage of the midbrain aqueduct at the end and route of microinjection cannot be entirely avoided, potentially exerting a direct effect on the spinal cord terminals (Supplementary Fig. 2D). This phenomenon has found some support in the literature with conflicting conclusions. Microinjection of Nα1 receptor antagonists has been shown to reduce medial prefrontal cortex (mPFC) hypersensitivity, whereas α2-AR antagonists are ineffective [23, 46]. Conversely, other studies have demonstrated that the α2-AR agonist clonidine has an analgesic effect in the mPFC of neuropathic rats [47]. We hypothesize that microinjections of clonidine in the mPFC may directly enter the cerebrospinal fluid circulation, leading to the inhibition of spinal cord receptors. Moreover, it should be noted that previous investigations primarily employed the intraperitoneal or intravenous administration of clonidine or yohimbine. Furthermore, while clonidine demonstrates a certain degree of therapeutic efficacy for a subset of individuals with headache in clinical settings, our research findings also indicated that its application in the LC of mice shows promising intervention effects for ASD-induced headache. Additionally, it also effectively mitigated the sleep disturbances caused by NTG-induced migraine-like headache. However, the response to clonidine differs among different types of headaches. Noradrenergic neurons have seven primary nuclei and are sporadically distributed in other areas, and subsequently, their functions differ. Therefore, based on fundamental research and clinical drug usage, systemic administration methods such as intraperitoneal delivery indeed struggle to achieve effects that specifically alter the function of a particular nucleus. In conclusion, precise targeted drug therapy is needed to realize effective, specific treatment for different types of headaches and sleep disturbances.

Notably, recent studies have elucidated the complex function of the LC. A small number of studies have demonstrated that the involvement of LC noradrenergic neurons in pain regulation is bidirectional [48]. Most studies have indicated that the projection of the LC into the spinal cord primarily inhibits pain perception, while the upward projection may increase pain sensitivity. For instance, Hirschberg [49] found that the chemogenetic activation of the pathway from the LC to the prefrontal cortex increases pain sensitivity, while activation of the pathway from the LC to the spinal cortex produces analgesic effects. Furthermore, Hickey [50] demonstrated that optogenetic activation of different regions within the LC exerts a bidirectional regulatory effect on heat pain: when dorsal cells in the LC are activated, thermal hyperalgesia increases, whereas a reduction is observed when the ventral cells are activated. Even in headache research, LC activation exhibits an opposite effect to that of the cortex and trigeminal nucleus, with LC destruction increasing cortical susceptibility to cortical spreading depression (CSD) while inhibiting trigeminal nucleus activity [22]. Schwarz [51] conducted a quantitative analysis of the input and output of LC noradrenergic neurons within the LC using a virus tracer method and found that neurons receiving similar fiber inputs project to different brain regions. These findings suggest that differences in anatomical location and projection specificity may underlie the differences in the effects of LC noradrenergic neurons. Additionally, the traditional concept of the homogeneous and global activation of the LC has recently been challenged by the identification of discrete activation modes. These modes involve different modules of noradrenergic neurons operating independently in terms of circuit function within their respective efferent domains, with distinct populations of cells assuming specific responsibilities [52]. Furthermore, there is evidence suggesting that the same group of cells can assume different roles [53]. Therefore, noradrenergic neurons in different parts of the LC may have different functions in pain, and even noradrenergic neurons in the same region may have different effects. Most importantly, the mechanism underlying pain itself is highly intricate, with significant differences in the pathways and neural circuits involved in head and facial pain compared to body pain. Additionally, acute pain and chronic pain exhibit distinct changes in pathways and loops, while mechanical pain and thermal pain are also associated with different neuroanatomical bases. Therefore, the investigation of the interaction between pain and sleep necessitates a case-by-case approach.

In conclusion, epidemiological studies provide compelling evidence to support the association between migraines and sleep disorders [7]. However, the mechanisms responsible for this association remain poorly understood, despite the presence of shared clinical conditions and anatomical pathways. Although significant progress has been made in recent years through various studies, further research is necessary to better understand this topic. It is crucial to explore the anatomy and neurotransmitters involved in this relationship, as it would not only offer valuable insights into the underlying mechanisms but also have implications for advancing our knowledge of migraine pathology and developing novel therapeutic approaches [7]. Our study specifically focused on the connection between acute headache and acute sleep disturbance. Through our experiments, we found that activation of LC noradrenergic neurons increased pain perception and sleep latency, while inhibiting LC noradrenergic neurons did not significantly affect pain sensitivity in normal mice. However, it did reduce sleep latency and improve sleep quality in mice with acute hyperalgesia. This highlights the important role played by LC noradrenergic neurons in bridging the gap between acute headache and acute sleep disorders. Our study did not differentiate between the upstream and downstream functions of the LC. Additionally, although we can infer from previous studies that sleep disorders are caused by headache induced by NTG, research related to the direct effects of NTG on sleep brain regions is lacking. Therefore, while this study provides a solid foundation for further investigation into the complex interaction between headache and sleep, future experiments must be carefully designed to further explore the specific mechanisms underlying the association between acute sleep disorders and acute headache.

### Supplementary Information

**Additional file 1**: A Schematic depiction of the timeline demonstrating the aggravating influence of ASD on NTG-induced behavioral changes (VEH, *n* = 12 mice; ASD, *n* = 12 mice; NTG, *n* = 12 mice; NTG+ASD, *n* = 12 mice). B, C and D Graphical representations of B, C and D demonstrate behavioral statistical trends recorded at the 26-hour time point within the timeline (1.5 to 2 hours post NTG injection). E and F In mice given intraperitoneal injections of NTG at 1-week intervals, there were no statistically significant changes in the periorbital (E) or paw (F) threshold.

**Additional file 2**: A Representative images illustrating the changes in LC neurons infected by two chemogenetic viruses 1.5 hours after CNO injection. B and C The viral infection itself, in the absence of CNO, does not have a significant effect on the pain threshold. D A representative image shows the location of the cannula and the arrival of the drug.

## Data Availability

All data, reagents, resources, and protocols are available from the corresponding author upon reasonable requests.

## Abbreviations

ASD: Acute sleep deprivation
CLON: Clonidine
CNO: Clozapine-N-oxide
CON: Control group
Cre: Cyclization recombinas
DBH: Dopamine-β-hydroxylase
EEG: Electroencephalography
EMG: Electromyography
LC: Locus Coeruleus
mPFC: Medial prefrontal cortex
NTG: Nitroglycerin
VEH: Vehicle
YO: Yohimbine
